# Adaptive Design of Fluorescence Imaging Systems for Custom Resolution, Fields of View, and Geometries

**DOI:** 10.34133/bmef.0005

**Published:** 2023-01-13

**Authors:** Roujia Wang, Riley J. Deutsch, Enakshi D. Sunassee, Brian T. Crouch, Nirmala Ramanujam

**Affiliations:** ^1^ Department of Biomedical Engineering, Duke University, Durham, NC, USA.; ^2^ Department of Pharmacology and Cancer Biology, Duke University Medical Center, Durham, NC, USA.

## Abstract

*Objective and Impact Statement:* We developed a generalized computational approach to design uniform, high-intensity excitation light for low-cost, quantitative fluorescence imaging of in vitro, ex vivo, and in vivo samples with a single device. *Introduction:* Fluorescence imaging is a ubiquitous tool for biomedical applications. Researchers extensively modify existing systems for tissue imaging, increasing the time and effort needed for translational research and thick tissue imaging. These modifications are application-specific, requiring new designs to scale across sample types. *Methods:* We implemented a computational model to simulate light propagation from multiple sources. Using a global optimization algorithm and a custom cost function, we determined the spatial positioning of optical fibers to generate 2 illumination profiles. These results were implemented to image core needle biopsies, preclinical mammary tumors, or tumor-derived organoids. Samples were stained with molecular probes and imaged with uniform and nonuniform illumination. *Results:* Simulation results were faithfully translated to benchtop systems. We demonstrated that uniform illumination increased the reliability of intraimage analysis compared to nonuniform illumination and was concordant with traditional histological findings. The computational approach was used to optimize the illumination geometry for the purposes of imaging 3 different fluorophores through a mammary window chamber model. Illumination specifically designed for intravital tumor imaging generated higher image contrast compared to the case in which illumination originally optimized for biopsy images was used. *Conclusion:* We demonstrate the significance of using a computationally designed illumination for in vitro, ex vivo, and in vivo fluorescence imaging. Application-specific illumination increased the reliability of intraimage analysis and enhanced the local contrast of biological features. This approach is generalizable across light sources, biological applications, and detectors.

## Introduction

Fluorescence imaging is one of the most widely used technologies for biomedical research. By leveraging exogenous or endogenous fluorophores, fluorescence imaging provides important information related to molecular and cellular activities, physiological functions, disease morphology, and biological structures in both preclinical research and clinical applications across biological model systems (in vitro, in vivo, and ex vivo). In preclinical research, fluorescence imaging is often conducted by large, centralized optical systems to produce high-quality images in terms of resolution, signal, and pixel depth. These systems are capable of being used for a variety of laboratory studies on the length scale of micrometers. For example, intensity-based techniques such as Förster resonance energy transfer are used widely with confocal microscopy for studying molecular interactions of proteins in vitro [1]. Fluorescence lifetime imaging microscopy, on the other hand, has been designed for studying cellular metabolism [2]. High-throughput fluorescence imaging systems [3] have been designed for drug screening and characterization of treatment response [3,4].

For preclinical applications, including vascular [5], anatomical [6,7], and molecular imaging [8–10], the design of existing laboratory systems must be extensively modified for different fields of view (FOVs), spatial resolutions, and different types and levels of contrast. In the case of clinical applications, imaging systems must also account for constraints such as time, space, cost, and ease of use [11,12]. In addition to the design considerations mentioned above, another crucial consideration for both applications is to achieve uniform illumination across the desired FOVs. Since fluorescence signal is proportional to the excitation illumination, uniform illumination is essential for quantitative intraimage analysis. Furthermore, when developing low-cost, high-fidelity imaging devices, it is important to consider maximizing contrast for weak fluorescent signal in tissues and minimizing the power budget by tailoring the illumination to efficiently use the available light output within the desired FOV [13,14]. Being able to incorporate all design considerations into one system can enable the system to work in different environments. Therefore, designing a single system to evaluate anatomical, functional, and molecular features across different types of samples for use in both the laboratory and the clinic by specialists or community health providers would broaden the opportunities for a wide range of translational applications.

We developed a computational method for the custom design of fluorescence imaging systems. Our algorithmic approach allows for a fixed number of sources and a single detector to generate uniform light for imaging across biological samples of specified sizes and geometries, whether it is for in vitro, ex vivo, or in vivo imaging*.* We have demonstrated the power of the computational model by designing a portable imaging system called the CapCell to perform brightfield and fluorescence imaging across different FOVs in both a wide-field and high-resolution format. We have designed the CapCell to achieve high- and low-aspect-ratio illumination patterns, using our computational method to determine the spatial placement of each light source. High-aspect-ratio imaging is used to demonstrate repeatable intraimage analysis of the entire length of core needle biopsies stained with a molecular indicator for HSP 90 expression while providing additional detailed visualization of local features across the biopsy. This design will allow us to perform rapid, wide-field imaging for the diagnosis of breast cancer biopsies. In the context of breast cancer diagnostics, our molecular imaging approach could also be applied to resected tumor margin assessment. In the realm of cancer diagnostics, other groups are similarly developing fluorescent molecular imaging methods for disease sites, including the brain [15], the gastrointestinal tract [16], and the ovaries [17]. In addition to uniformity, a low-aspect-ratio illumination pattern maximizes power density with efficient use of excitation light and thus enhances the contrast of metabolic indicators in preclinical mammary window chamber tumors [18] and breast tumor organoids. We are also interested in applying this work to preclinical metabolic imaging of in vivo tumors, to observe tumors at multiple resolutions through regression and recurrence. The CapCell can be modified for each of these applications, allowing for a single system to image across anatomical locations and sample types of various sizes.

## Results

### Global optimization to design a fluorescence imaging system with uniform illumination

**Fig. 1.**
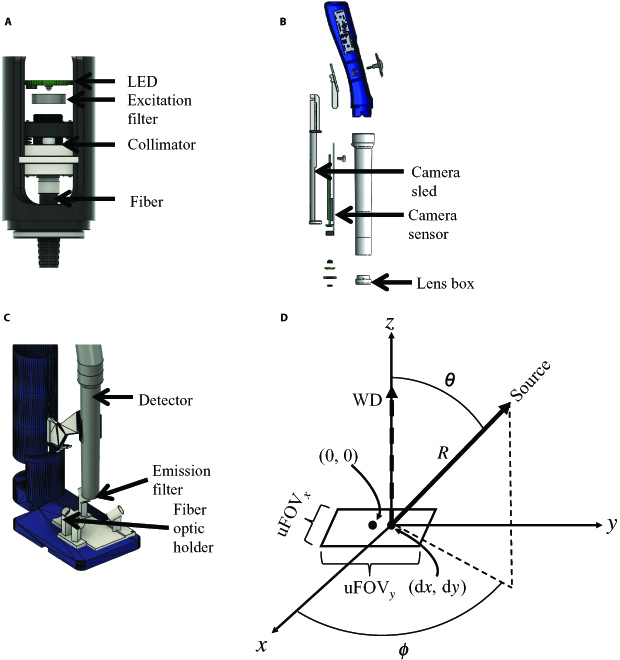
3D models and schematic for the imaging system designed for these studies. (A) Optical components for collimating LED emissions into the optical fiber bundle. (B) An exploded view of the Pocket Colposcope used as a detector in this system. A 5-megapixel CMOS camera sensor is mounted to the camera sled. Moving the sled changes the distance between the sensor and the lens box, modifying the system’s working distance and field of view. (C) A model of the combined microscope platform. The detector and optical fibers are held in position with a 3D-printed stage. The detector can be translated vertically to adjust the field of view and resolution of the system. The fiber optic holder component is replaceable, allowing for multiple fiber configurations. (D) Key variables include the angle between the axis of the detector and the fiber (*θ*) and the radial distance from the tip of the fiber to the sample plane (*R*). Additional variables for optimization include the angle between the fiber and the positive *x* axis about the *z* axis (*ϕ*), and a shift in the center of the beam profile in the *x* and *y* directions (d*x*, d*y*) from the origin (0,0). Inputs to the model include the dimensions of the desired uniform field of view (uFOV*_x_*, uFOV*_y_*), the number of sources, and the upper and lower bounds for each optimized variable (*R*, *θ*, *ϕ*, d*x*, d*y*). The working distance from the detector to the sample is included (WD) but does not impact optimization.

The CapCell consists of an optimized illumination geometry and a color detector. Figure 1A illustrates the optical setup for the fiber-based illumination, consisting of a light-emitting diode (LED), an excitation filter, a collimator, and an optical fiber. The detector part (Fig. 1B) consists of a previously described, low-cost digital colposcope (Pocket colposcope) [13] and an emission filter. The illumination and the Pocket detector are assembled on a 3D-printed microscope stage as shown in Fig. 1C. For the illumination optimization, we developed a generalizable computational algorithm to determine the placement of several individual sources to flexibly maximize contrast and the power efficiency of a fluorescence imaging system. For the purposes of illustration, we designed 2 different illumination distributions for 2 distinct uniform fields of view (uFOVs): One with a high aspect ratio (uFOV*_x_* >> uFOV*_y_*) and another with a low aspect ratio (uFOV*_x_* = uFOV*_y_*). Each fiber was modeled as a Lambertian emitter (Fig. 1D), and the position of each source was optimized using a global optimization algorithm. We modeled the set of 4 illumination fibers used in the imaging system. The emission half-angle for each fiber was estimated by performing a least-squares fit between a fluorescent standard image with known spatial positions and a simulated illumination distribution. The final simulated illumination distribution was found by summing the contributions of each individual fiber. Our optimization algorithm aimed to optimize 5 spatial coordinates for each fiber: the spatial shift from the origin (d*x* and d*y*), the radial distance between the source and the sample (*R*), the angle between the *z* axis and the *xy* plane (*θ*), and the angle between the fiber and the positive *x* axis (*ϕ*). Uniformity was assessed with a linear combination of a mean–max ratio (mean value/max) and an efficiency term representing the ratio of illumination inside and outside of the desired uFOV. We first ran optimizations using the coefficient of variation (variance divided by mean) as previously demonstrated for characterizing image uniformity [19–21]. While this cost function can select for uniform illumination, the illumination profile generated by the algorithmic solution is not confined to the desired uFOV, resulting in a decreased power density seen by the sample. A second cost function, the mean–max ratio was also tested for optimization. It was expected that as the mean value approached the max, the optimization would converge on a solution where most pixels had a similar value near the mean. To further increase the efficiency of optical power used, an efficiency term was added that favored maintaining power density within the desired uFOV. These results are summarized in Fig. S1A. Here, the optimization utilizing the combined mean–max and efficiency term generated uniform illumination within the uFOV with a ~4 to 5× increase in power density. The uFOV was modified to generate illumination distributions with different aspect ratios. A high-aspect-ratio uFOV of 4 × 12 mm was selected for breast biopsy imaging, and a low-aspect-ratio uFOV of 6 × 6 mm was selected to accommodate the average size of solid tumors imaged in murine window chambers and breast tumor organoids.

To demonstrate the convergence and repeatability of this approach, optimization for each aspect ratio was run 5 times. The resulting illumination distributions are shown in Fig. S2. For each case, the algorithm produces a similar desired uniform illumination distribution, with power density maintained within the uFOV. Optimization of the high-aspect-ratio uFOV ran for an average of 94 s and required an average of 32,000 iterations to converge. Optimization of the low-aspect-ratio uFOV ran for an average of 7 s and required an average of 23,000 iterations to converge. For each trial, optimization was terminated once the mesh size was within a default tolerance of 10^−6^.

The experimental system to validate the illumination distribution for these 2 aspect ratios included a blue LED (470 ± 13 nm), an excitation bandpass filter (466 ± 20 nm), 4 optical fibers (core diameter = 600 μm; numerical aperture [NA] = 0.39), and a central imaging system comprising a lens box and complementary metal-oxide semiconductor (CMOS) detector with a 535 ± 21 nm emission bandpass filter. To represent the physical fibers in silico, the viewing half-angle of each fiber was estimated to be 6.09°, 6.29°, 6.08°, and 6.00°. Uniformity of the resulting irradiance distribution was assessed by imaging a standard fluorescence slide illuminated for the 2 different configurations of the optical fiber placements, designed for low and high aspect ratios. The optimized spatial parameters are listed in Table for the low- and high-aspect-ratio distributions.

**Table:** Final parameter outputs after optimizing each fiber for a high-aspect-ratio and a low-aspect-ratio illumination density.

Variable	High aspect ratio (4 × 12 mm)				Low aspect ratio (6 × 6 mm)			
	Fiber 1	Fiber 2	Fiber 3	Fiber4	Fiber 1	Fiber 2	Fiber 3	Fiber 4
*R* (mm)	23.43	20.00	20.25	22.28	12.72	13.30	12.53	12.52
*θ* (°)	31.33	38.31	58.22	46.94	47.38	45.24	46.03	45.00
*ϕ* (°)	57.66	160.15	268.53	287.37	2.12	95.80	181.40	270.32
d*x* (mm)	0.03	−0.06	−0.28	0.35	0.85	−1.33	−0.68	1.99
d*y* (mm)	1.12	4.75	−2.96	−3.11	1.74	1.04	−1.51	−0.65

**Fig. 2.**
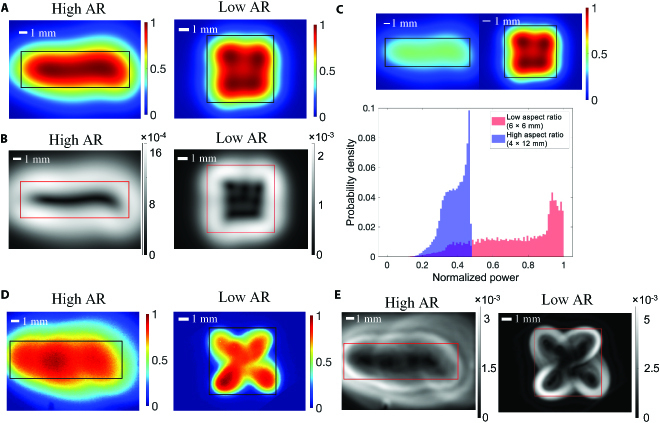
Computational optimization can design application-specific illumination that is uniform and optical power-efficient. (A) A simulated illumination distribution for a high-aspect-ratio (AR) (uFOV*_x_* = 4, uFOV*_y_* = 12) and low-AR (uFOV*_x_*=6, uFOV*_y_* = 6) scenario. All optimizations were run for a maximum of 50,000 evaluations with a mesh tolerance of 1 × 10^−6^ and a step tolerance of 1 × 10^−6^. The angular position, θ, was bounded between 30° and 60° and radial distance *R* was bounded between 12.5 and 40 mm. (B) Resulting images after calculating the gradient of each image from (A). (C) Normalized histograms of the simulated image from (A) and (B). Both histograms were normalized to the maximum intensity of the low-AR image. Images after this normalization are included to visualize the differences in power. Pixels were only included from within the desired uniform field of view. (D) Benchtop images of illumination systems translated from simulation coordinate data. (E) Gradient images calculated from the images in (D).

Figure 2A shows the illumination distributions created by global optimization for the high- and low-aspect-ratio scenarios. A black box indicates the input uFOV for each case, and each image is normalized to its own maximum value. To demonstrate the uniformity within each scenario, the gradient of each image was calculated using MATLAB’s built-in gradient function. Figure 2B shows that the central region is darkest in both images, corresponding to a near-zero gradient and minimal signal change across the uFOV. In addition to uniformity, the computational optimization platform maximizes power density. To compare the relative intensity of the 2 illumination distributions, both distributions were first normalized to the maximum intensity of the low-aspect-ratio solution. Probability density functions were generated for each normalized image (Fig. 2C). The low-aspect-ratio solution exhibits a 1.9 increase in average power, suggesting that the algorithm can more efficiently fill the uFOV. Figure 2D shows benchtop images, and Fig. 2E shows their gradient calculations. Before calculating the gradient, images were smoothed with a Gaussian filter (neighborhood = 50) to remove noise associated with the detector and imperfections in the fluorescent standard. The gradient is lowest in the center of the uFOV.

We leveraged the optimized illumination to design a fluorescence imaging system, the CapCell. The CapCell is capable of imaging at multiple resolutions by varying the distance between the CMOS camera and the lens box at the distal end of the device. The CapCell achieves a linear dynamic range over the range of biologically relevant fluorophore concentrations for a given application. The dynamic range of the CMOS camera in the CapCell was assessed using a Stouffer 41-step transparent wedge (optical density, 0.1 to 4) and a white LED lamp. The average pixel intensity was calculated and plotted against the known light transmission of the filter for all integration times. Figure S3A shows the relationship between pixel intensity captured by the CMOS camera and known light transmission. Regression analysis of the dynamic range data shows strong linear correlations (*R*^2^ = 0.97). To assess the linearity of fluorescence measurements with the CapCell, we used a fluorescent Hsp90 probe (HS-27) that specifically labels breast tumor specimens [10,22,23]. We previously demonstrated that HS-27 can be used as a molecular imaging probe for breast cancer detection in vivo and ex vivo [24]. Imaging phantoms were created with tissue-mimicking optical properties and known concentrations of HS-27 (0–25 μM based on prior preclinical and clinical studies [25]). Polystyrene beads were used to mimic the optical scattering (μs′  = 20 cm^−1^) of 4T1 mammary tumors [26]. Images were acquired using the high-aspect-ratio illumination platform. Figure S3B shows the average pixel intensities of the tumor phantoms for each of the 6 concentrations, indicating a strong linear correlation.

### CapCell supports spatial analysis of fluorescence in ex vivo tumor tissue specimens

We aimed to demonstrate that the uniform illumination distribution generated by our computational optimization design platform increased the repeatability of fluorescence imaging compared to unoptimized, nonuniform illumination. An ex vivo biopsy study was performed to demonstrate that the fluorescence signal from the high-aspect-ratio optimized setup varied less compared to an unoptimized setup. Here, nonuniformity was generated by translating the distance between the sample plane and fibers along the *z* axis by 6 mm, to a point where the illumination distribution was no longer uniform. The working distance (WD) between the detector and the sample plane was maintained to reduce confounding effects on sample intensity. 4T1 mammary tumors were grown in the fourth right mammary gland, and 2 biopsies were acquired randomly from each tumor. The specimens were stained with either 100 μM HS-27 (specific stain) or HS-217 (nonspecific stain) (*n* = 5 for each group) for 1 min and washed in phosphate-buffered saline (PBS). The biopsy was imaged under both optimized and unoptimized illumination at 3 different positions in the FOV at a WD sufficient to capture the entire sample in a single image (WD = 25 mm).

**Fig. 3.**
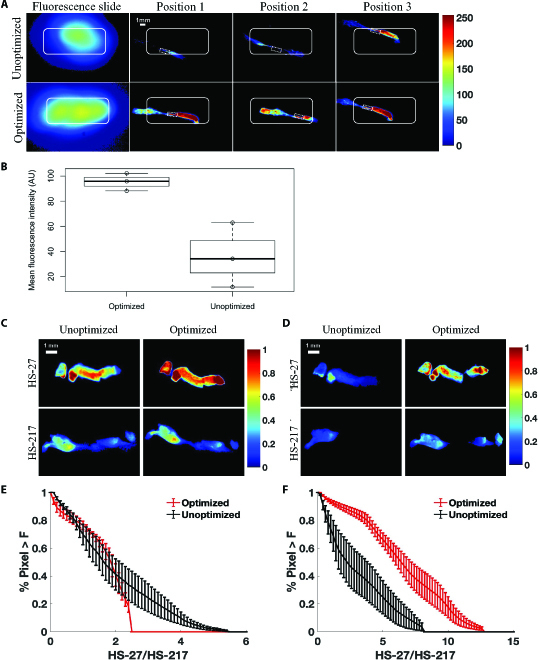
Comparison of optimized and unoptimized setups’ imaging repeatability at different positions in the field of view (FOV). (A) Representative wide-field fluorescence image of a fluorescence standard slide and an ex vivo murine breast cancer (4T1) biopsy specimen. Ex vivo 4T1 (murine mammary tumor) tissue was stained with either 100 μM HS-27 or HS-217 for 1 min prior to PBS washing and imaging. The biopsy was moved into 3 different positions in the FOV of both an unoptimized (top) and optimized setup (bottom). The white bounding box indicates the placement of the uniform illumination distribution. The corresponding area is also labeled in the unoptimized images. The optimized setup shows better consistency in the fluorescence signal within the bounding box compared to the unoptimized setup. The overlapping region of the biopsy is highlighted by the white, dashed rectangle. (B) Box plot of average fluorescence intensity of overlapping biopsy region (dashed) that lies within the bounding box at 3 different positions. The unoptimized setup has an average fluorescence ranging from 11.58 to 62.86 AU depending on its position within the illumination profile, which is much wider compared to the optimized setup with an average fluorescence intensity ranging from 88.15 to 102.20 AU. All images were 8 bits with a range of 0 to 255 AU. (C) Representative CapCell fluorescence images of 4T1 biopsies stained with HS-27 or HS-217 imaged by the wide-field mode using optimized and unoptimized setup. For all groups, *n* = 5 biopsies. (D) Representative CapCell fluorescence images of 4T1 biopsies stained with HS-27 or HS-217 imaged by the high resolution using optimized and unoptimized setup. For all groups, *n* = 5 biopsies. (E) Comparison of HS-27/HS-217 ratio survival curves of wide-field images acquired by optimized (red) and unoptimized (black) setups. There is no statistical significance of HS-27/HS-217 ratio between 2 setups. (F) Comparison of HS-27/HS-217 ratio survival curves of high-resolution images acquired by optimized (red) and unoptimized (black) setups. There is a statistically significant difference of HS-27/HS-217 ratio between 2 setups (*P* < 0.05). The optimized setup shows a high HS-27/HS-217 ratio. Survival curves show the mean ± SEM. Statistical significance between survival curves was determined by a Kolmogorov–Smirnov test.

Figure 3A shows representative fluorescence standard slide images and HS-27 fluorescence images of the same biopsy at 3 different positions. The top panel was imaged with the unoptimized setup, and the bottom panel was imaged using the optimized setup. The outlined region of uniform illumination is determined from a fluorescence standard slide image, and the region has less than 10% variance from the maximum fluorescent intensity in the FOV. The size of the uniform illumination is determined from the input desired FOV on the fluorescence standard slide image. The white bounding box in all images represents the uniform illumination region. Within the bounding box, the fluorescence images acquired by the optimized setup showed that salient features were visible regardless of position in the FOV compared to the unoptimized illumination. By comparing the mean fluorescence intensity of overlapped regions of biopsy (highlighted by a white, dashed rectangle in Fig. 3A) for all 6 images within the bounding box, the mean intensity of the optimized setup had a much tighter distribution with pixel values ranging from 88.15 to 102.2 arbitrary units (AU), while the unoptimized setup produced mean intensity values from 11.58 to 62.86 AU (Fig. 3B).

We also performed a simple flat field correction to the unoptimized setup to compare with our optimized setup. We divided the illumination pattern using an image of the fluorescence standard slide from the fluorescent biopsy image in a pixel-by-pixel manner. By performing this division, we aimed to correct the variation in fluorescent signals in the unoptimized setup caused by illumination nonuniformity. The effect of flat field correction on a representative biopsy stained with HS-27 is shown in Fig. S4. In the corrected unoptimized image, the fluorescence contrast was increased in the left part of the biopsy, but the fluorescence signals of the right part cannot be recovered as shown in the optimized setup, indicating that flat field correction alone is insufficient for recovering quantitative fluorescent values.

Additionally, we used an established protocol [22] to validate the imaging performance of the CapCell and compare the Hsp90 specificity between optimized and unoptimized setups. Murine breast tumor (4T1) biopsies (*n* = 5 per group) were treated with 100 μM HS-27 (specific stain) or HS-217 (nonspecific stain) for 1 min. Higher fluorescence signals are observed in HS-27-stained biopsies than in HS-217-stained biopsies for both optimized (Fig. 3C) and unoptimized systems (Fig. 3D) in wide-field images. However, in high-resolution images, the contrast between HS-27 and HS-217 diminished in the unoptimized system due to nonuniform illumination and lack of sufficient power density, while the optimized setup maintains the specificity of HS-27 fluorescence. Based on previous studies, Hsp90 specificity is defined as the HS-27/HS-217 ratio [22], where all pixels of HS-27 images are divided by the mean fluorescence intensity of all HS-217 images. Here, we compared the survival curves (1 − cumulative distribution function) of the HS-27/HS-217 ratio between the optimized high-aspect-ratio platform and unoptimized setup for both wide-field (Fig. 3E) and high-resolution (Fig. 3F) imaging. There is a statistically significant (*P* < 0.05) increase in probe contrast in the high-resolution image for the optimized setup compared to the unoptimized setup. Optimizing illumination uniformity and power density of the system enhances the image contrast between HS-27 and HS-217 samples at multiple resolutions.

**Fig. 4.**
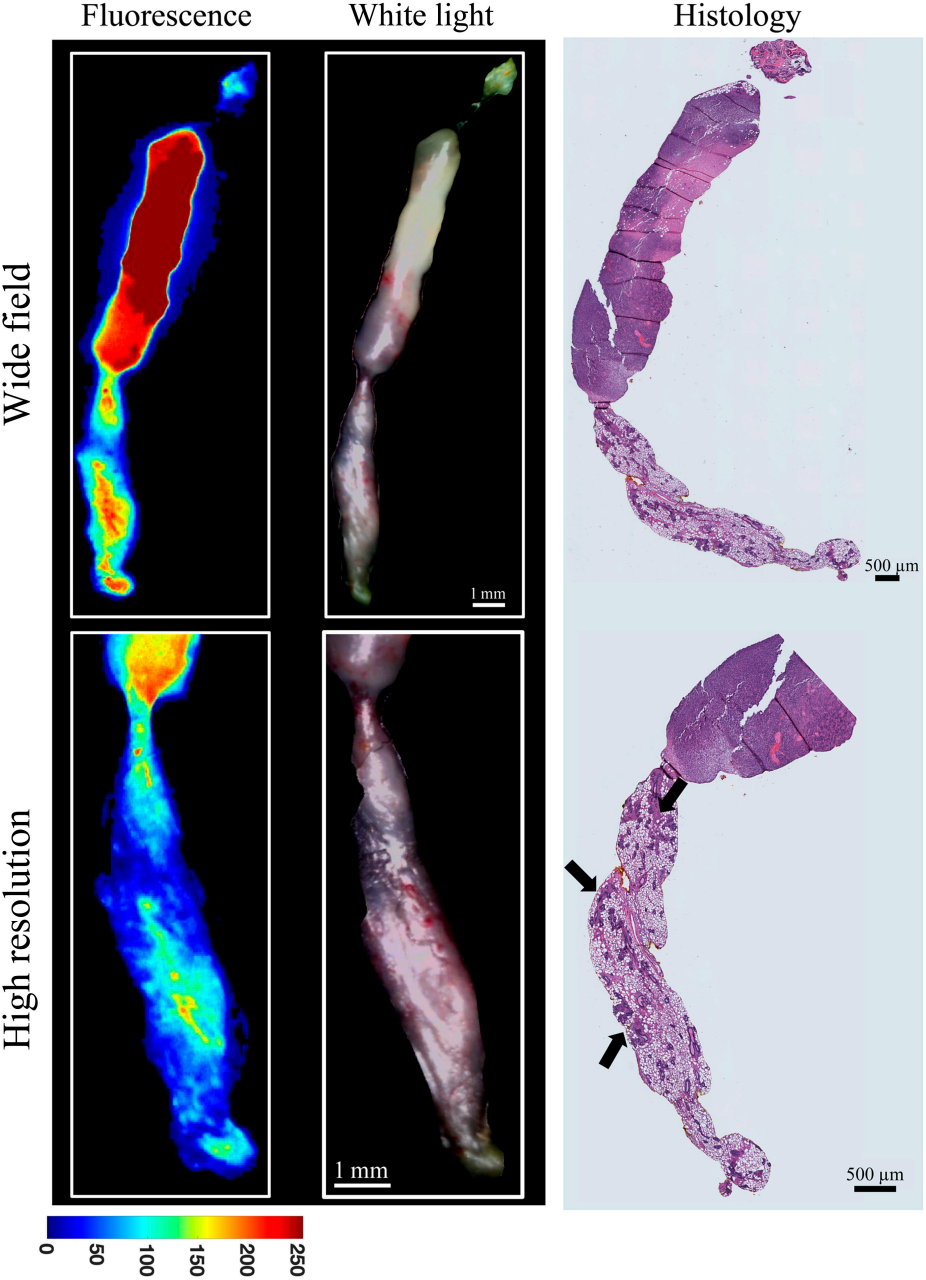
Coregistration of CapCell brightfield and fluorescence images of an ex vivo murine breast cancer (4T1) biopsy specimen acquired at wide-field and high-resolution imaging modes. Ex vivo 4T1 (murine mammary tumor) tissue was stained with either 100 μM HS-27 or HS-217 for 1 min prior to PBS washing and imaging. From left to right are the Hsp90 fluorescence images, white light brightfield images, and hematoxylin and eosin histology slide of the same biopsy specimen. The top panel consists of wide-field images, and the bottom panel consists of high-resolution images. The high-resolution images demonstrate more detailed structural features that correspond to the histology image. The black arrows in the bottom histology image indicate the small clusters of 4T1 tumor cells infiltrating from the tumor into normal adipose tissue. (The histology image was processed with Adobe Photoshop for rotational purposes only.)

The same biopsy study was performed to demonstrate the utility of high-resolution imaging for ex vivo samples. An HS-27-stained biopsy was imaged at both wide-field and high-resolution scales. After imaging, the biopsy was fixed in 10% formalin solution and sent to the Duke Research Immunohistology laboratory for histology analysis. The histology slide was scanned on a Zeiss Axio Imager Z2 Upright Microscope. Wide-field (WD = 25 mm), high-resolution (WD = 12.5 mm) fluorescence, and brightfield images captured by the CapCell are shown in Fig. 4. A 25-mm WD was used for wide-field imaging (resolution = 24.1 μm) to ensure the biopsy could be captured in a single FOV and provide concordance to the histology images. High-resolution imaging, at a 12.5-mm WD (resolution = 12.5 μm), captured more detailed features of the specimen. In the bottom panel of Fig. 4, the high-resolution image demonstrated more detailed fluorescence and morphological features that corresponded to histology and were otherwise unseen in the wide-field images. Small clusters of 4T1 tumor cells (indicated with the black arrows) within the adipose tissue shown in the histology slides can be seen in an increased fluorescent signal of the high-resolution images but not in wide-field images. The uniformity of our optimized setup enabled the recognition of local features in the larger biopsy image.

### Maximizing high power density for a given FOV allows for improved contrast with the CapCell

**Fig. 5.**
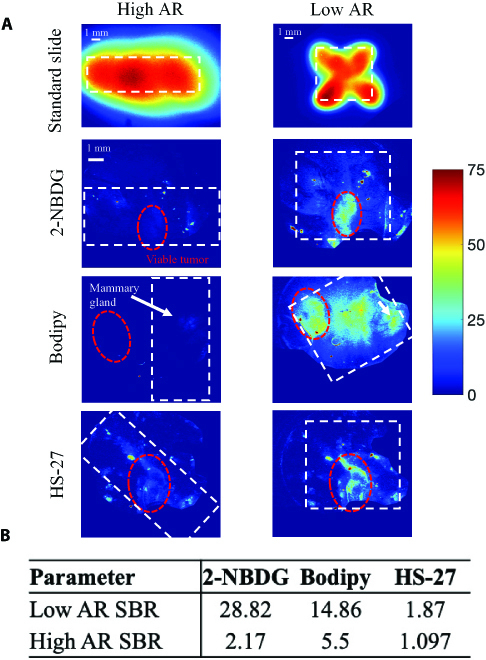
The low-AR CapCell setup demonstrates higher signal-to-background ratio (SBR) than the high-AR setup. (A) Fluorescence images standard slides and of an in vivo murine breast cancer (4T1) mammary window imaged after intravenous delivery of either 2-NBDG, Bodipy FL C16, or HS-27. Each fluorophore is imaged with the low-AR and high-AR illumination platforms. The viable tumor region is outlined by a red ring. The white dashed box indicates the position of the uniform illumination in both cases. A region of mammary tissue is also identified by a white arrow in the Bodipy images. All images were taken 60 min after intravenous injection of a fluorescent probe. (B) Table of the SBR of both the optimized setup and unoptimized setup for 3 different exposure times. The SBR is defined as the average intensity of the postinjection image divided by the average intensity of the preinjection image. SBR is a unitless metric. The optimized setup shows better SBR for all fluorophores.

We next aimed to show the impact of maximizing power density during the optimization process. To accomplish this, we imaged orthotopic 4T1 tumors grown in a window chamber with 1 of 3 fluorescent agents: 2-NBDG, a glucose analog; Bodipy FL C16, a fatty acid reporter; and HS-27, a reporter of Hsp90 expression. 4T1 mammary tumors were grown in the fourth right mammary gland of athymic nude mice. Once tumors reached an approximate size of 5 × 5 mm, the skin over the tumor was removed and replaced with a 12-mm-diameter titanium window and a glass coverslip. On the day of imaging, a background fluorescence image was taken using both the low-aspect-ratio and high-aspect-ratio illumination platforms. Animals then received a retro-orbital injection of either 2 mM 2-NBDG, 200 nM Bodipy FL C16, or 20 mg/kg HS-27. At the 60-min time point, fluorescence images were acquired with both illumination platforms. All images were manually masked to remove the titanium ring from the image. Sixty-minute postinjection images for all 3 fluorophores with either the low- or high-aspect-ratio illumination platform are shown in Fig. 5A and the corresponding signal-to-background ratio is in Fig. 5B.

For each image, the tumor region is indicated by a red circle. The approximate region of uniform illumination is depicted by a white dashed box. 2-NBDG signal and HS-27 signals both increase most within the tumor, in line with the highly glycolytic phenotype of 4T1 tumors and previous studies of HS-27 intravital imaging [24]. The 60-min images illustrate the loss of contrast associated with the lower power density of the high-aspect-ratio illumination platform. By placing the high-aspect-ratio illumination vertically over the mammary gland, we demonstrate that only the highest power density region of this platform can generate a signal in this application. Preinjection and 60-min postinjection images were used to calculate the signal-to-background ratio for each fluorescent probe with each illumination platform. By changing the spatial positions of the fiber sources, it was possible to increase the level of contrast across the entire region of interest, demonstrating the flexibility of our computational optimization platform. We observed that for all 3 fluorophores, signal-to-background ratio increased when the low-aspect-ratio illumination platform was used (Fig. 5B).

**Fig. 6.**
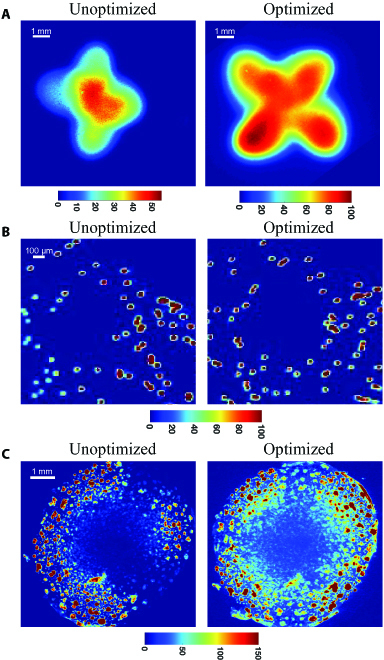
The CapCell can be applied to in vitro studies. (A) Representative fluorescence standard slide images of the unoptimized (left) and optimized (right) low-aspect-ratio illumination platform. (B) Representative 1:5 diluted fluorescent 45-μm bead images of the unoptimized (left) and optimized (right) low-aspect-ratio illumination platform. (C) Representative fluorescence images of one 4T1 organoid culture stained with Bodipy FL C16, a fluorescent reporter of fatty acid uptake imaged with the unoptimized (left) and optimized (right) low-aspect-ratio illumination platform. The sample was incubated with 1 μM Bodipy FL C16 for 2 h prior to imaging.

Figure 6 demonstrates that the CapCell is useful for in vitro studies, specifically to image tumor organoids. We first performed a fluorescence bead study to mimic in vitro applications. A 45-μm yellow green fluorescent microsphere solution was diluted in a 1:5 ratio using PBS. A droplet of the diluted solution was imaged by both an unoptimized and an optimized platform. The unoptimized scenario was created by changing the offset of each fiber (d*x*, d*y*) to 0 to ensure that the centers of all individual illumination distributions overlap. Figure 6A shows the fluorescence standard slide images captured by either setup. The optimized image has a relatively larger illumination size and region of uniform illumination. Figure 6B demonstrates that the fluorescence signal of the optimized image (right) is more consistent across different fluorescence beads compared to the unoptimized setup. We used the CapCell and the low-aspect-ratio illumination platform to image one unstained 4T1 organoid sample and one 4T1 organoid stained with Bodipy FL C16. The stained organoid was also imaged with either optimized or unoptimized illumination (Fig. 6C). Because the organoid specimen had a dome structure with a thicker center compared to the edges, we observed lower fluorescence signals from the center compared to the edges. However, when looking at the edges of the fluorescence signals, the optimized setup demonstrates improved signal consistency compared to the unoptimized setup, where more variance was observed in the lower right corner of the FOV shown in Fig. 6C.

## Discussion

We have developed a computational optimization algorithm to generate illumination distributions that are uniform, have a high-power density, and minimize the amount of excitation light that falls outside of the desired FOV. Furthermore, we leveraged this approach to develop the CapCell to image across different biological systems and at different resolutions without the need for multiple devices. We simulated each source in the device as a Lambertian emitter and used a genetic pattern search global optimization algorithm [27] for the spatial positioning of the illumination fibers. Our optimization approach can be applied to any detector and can accommodate a wide variety of sources to image a multitude of different biological systems.

Uniform illumination is a necessary element to reliably and repeatably capture quantitative fluorescence images [28] in an effort to standardize the technique for both clinical and preclinical studies. Conventional methods for generating uniform illumination include Köhler illumination [29], point scanning [30], refractive optical components to generate a flat-top profile [31], or, alternatively, a multitude of optical fibers in an annular geometry [32]. Additionally, flat field correction is a common postacquisition processing technique to compensate for imperfections in illumination source uniformity [33,34]. While an important step for ensuring reliable quantitative imaging, flat field correction is limited in its ability to overcome low contrast, increased noise, and finite dynamic range [35]. This is particularly relevant in the design of low-cost or point-of-care fluorescence imaging systems where finite dynamic range and reduced sensitivity are a concern. Previously designed low-cost systems have involved either adding fluorescence excitation with a single LED focused by a lens to produce a Gaussian profile [36,37] or by placing multiple excitation sources concentrically about the detector [38,39]. We have demonstrated that our technology is able to improve the analysis of fluorescence images compared to realistic illumination profiles implemented in these other low-cost imaging systems. The validation of our system is akin to other works [31,32,40] aimed at generating uniform illumination by augmenting the Gaussian profile of a standard laser. We demonstrate a repeatable, high-contrast quantitative fluorescence system using a detector that would otherwise not be suitable for such imaging over multiple sample types.

Previously developed computational models have been used to design uniform illumination specific to human observation, such as room lighting, where the FOV is large and the required power density is not a notable concern. These methods employ a number of optimization approaches to solve for uniformity, including analytical methods [41,42], signal processing approaches [43,44], and global optimization algorithms such as simulated annealing [19]. Our method has been designed for a more challenging application, fluorescence imaging. Fluorescence microscopy is often photon-starved and focused on a relatively small FOV [45]; we address this with a custom cost function to weigh both uniformity and power density. Other groups have leveraged deep learning methods to optimize laser power for multiphoton microscopy [46] or to design illumination patterns for Fourier ptychographic microscopy [47]. Our approach offers a computationally inexpensive alternative to rapidly generate uniform illumination profiles with sufficient power density for fluorescence applications.

Lambertian emitter illumination models have been previously shown to account for spherical coordinates at large distances from the observer [42]. We demonstrate that fiber illumination can be fairly represented as a Lambertian emitter for this application, and we are able to translate the results of our simulation to matching benchtop illumination. However, the divergence between the simulation and benchtop was greater for the low-aspect-ratio design, where all fibers were positioned at a closer radial distance. Therefore, it is possible that at close WDs, optical fibers deviate from the Lambertian emission assumption. To increase concordance between simulation and benchtop, we will consider implementing a Gaussian model of fiber illumination [48] in future generations that can account for spherical coordinate positioning.

The implementation of a genetic pattern search algorithm was successful in finding an optimal result. This approach combines 2 established optimization algorithms: genetic algorithm [49] and pattern search [50]. Genetic algorithms are a stochastic class of algorithms capable of quickly scanning a parameter space for a local minimum. Conversely, pattern search is a nonrandom numerical approach well-suited for converging on a stationary point [51]. It has been demonstrated that the combination of these 2 algorithms in one genetic pattern search algorithm is able to converge better on a global minimum [27,52]. While our optimization did not converge on a single optimal solution, we demonstrated that this approach was able to converge on a family of solutions. From an engineering perspective, any output solution could be implemented to achieve the desired imaging results since each solution fits the desired criteria of uniformity and high-power density. The variability introduced by the stochastic element of the genetic algorithm is one limitation of our proposed solution. Future work can be done to tune hyperparameters (such as pattern size and rate of expansion) of this algorithm in order to increase efficiency and ensure that an optimal solution is found [27]. Novel to this work was the focus on designing illumination with a limited number of illumination sources and power budget. Previous publications have included a multitude of light sources, with the goal of designing illumination suitable for visual observation in daily settings [41,42,44,53,54]. Given the need for high power density to achieve a resolvable fluorescent signal, previous approaches could not be directly translated to fluorescence imaging applications. In considering light efficiency, we expand the application of computational optimization to consider scenarios requiring high power density, such as fluorescence imaging.

With our current proof-of-concept system, we demonstrate that our computational approach is able to design custom illumination for different FOVs required to image breast core needle biopsies, murine window chamber models, and organoids. While we demonstrated 2 distinct optimization solutions for geometries relevant to our applications, optimization could be rerun to accommodate samples of any geometry. The outputs of our computational method could be implemented in several ways. A variety of templates could be used to generate the desired illumination profile for each biological application, akin to switching filters in a microscope. The system could be automated, with an actuator moving each light source to its computationally determined position, or an array of illumination sources could be embedded in a single device, with only a subset turned on to generate the desired illumination profile similar to other devices [55,56]. The flexibility in the approach to achieve different illumination profiles is important for a device to be relevant for the myriad biomedical imaging applications, including benchtop experiments, fieldwork, development of translational methods, and clinical imaging. Using the same imaging system across applications and biological systems can help maintain data consistency when comparing research results across cells, organoids, and tissue imaging, both ex vivo and in vivo. This allows for continuity in data collection from preclinical to translational work across all scales, and it fills a need for accessible fluorescence imaging tailored to the application.

## Materials and Methods

### Experimental and technical design

The objective of this study was to establish and demonstrate a computational method for designing uniform illumination. The first set of studies was done in silico and at the benchtop to demonstrate that a computational approach could generate the desired illumination distributions and that those distributions could be translated to benchtop devices. Subsequent studies demonstrated the benefit of imaging with uniform illumination over nonuniform illumination. Preclinical biopsy samples were stained with a tumor-specific fluorescent probe and imaged to demonstrate the increased reliability of intraimage analysis when using uniform illumination. Murine window chamber studies demonstrated the importance of generating a unique illumination distribution for each sample geometry. Here, the image contrast generated by 2 different uniform illumination distributions was compared. Finally, tumor-derived organoids were imaged to demonstrate an application of imaging in vitro samples under uniform illumination conditions.

### Design of the CapCell

The CapCell consists of 2 components: the optimized illumination and a consumer-grade color CMOS detector. We used a 0.40 NA fiber collimator (Edmund Optics) to collimate a 470 ± 13 nm LED driven at 1 A into a 1:4 fan out optical fiber bundle (core diameter = 600 μm; NA = 0.39) (Thorlabs) (Fig. 1A), filtered with a 466 ± 20 nm bandpass excitation filter (Edmund Optics). The detector (Fig. 1B) consisted of a previously described, low-cost digital colposcope (Pocket colposcope) [13] and a 535 ± 21 nm emission filter (Edmund Optics). Briefly, the device consists of a 5-megapixel CMOS sensor and a 5-prime lens box. The sensor can be translated to change the distance between the lens box and the sensor and consequently the resolution of the system. A concentric ring of white LEDs allows the Pocket to acquire brightfield images. The fiber sources and Pocket are held in position using a 3D-printed microscope stage (Fig. 1C).

### Computational model of source illumination

Figure 1D depicts the modeled illumination system. Four light sources are placed about a sample in a spherical coordinate system. Spatial positioning is defined with the radial distance between the source and sample plane (*R*), the angle formed between source and the positive *z* axis (θ), and the angle between the source and the positive *x* axis (*ϕ*). A shift in the central point of source illumination is modeled with (d*x*, d*y*), and the region of desired uniformity is defined by (uFOV*_x_*, uFOV*_y_*). The detector is placed at a WD from the sample. All sources are modeled as Lambertian emitters [19,44], an exponential cosine function.

$$E(r,\theta )=E_{0}(r)\cos^{m}(\theta )$$ (1)

where *E*_0_(*r*) is the irradiance distribution on-axis at a distance *r* from the source, θ is the viewing angle, and *m* is a value derived from the viewing half-angle by:

$$m=-\frac{\ln(2)}{\ln(\cos(\theta_{1/2}))}$$ (2)

θ_1/2_ represents the viewing half-angle, the viewing angle at which the irradiance decreases by half the intensity at θ = 0°. Rewriting Eq. 1 with spherical coordinates yields:

$$E(x,y,z;R,\theta ,\phi )=\frac{{\left[(x-R\sin\theta \cos\phi )\sin\theta \cos\phi +(y-R\sin\theta \sin\phi )\sin\theta \sin\phi +(z-R\cos\theta )\cos\theta )\right]}^{m}}{{\left[{\left(x-R\sin\theta \cos\phi \right)}^{2}+{\left(y-R\sin\theta \sin\phi \right)}^{2}+{\left(z-R\cos\theta \right)}^{2}\right]}^{(m+2)/2}}$$ (3)

The total irradiance distribution can be found as the sum of individual sources (*N*) after normalizing by the average intensity of the source at a small value of *R*:

$$E_{\mathrm{net}}=\sum_{n=1}^{N}E_{n}\frac{\text{Mean}(E_{n})}{\text{Mean}(E(x,y,z;5,0,0)}$$ (4)

For optimization, *E*_net_ was only calculated over the region defined by (uFOV*_x_*, uFOV*_y_*).

### Global optimization of illumination distribution

Genetic pattern search optimization was implemented using a pattern search function of the Global Optimization toolbox of MATLAB 2021A (MathWorks) to determine the spatial coordinates for each of the 4 simulated fiber sources. The algorithm optimized *R*, θ, *ϕ*, d*x*, and d*y* for each fiber for a total of 20 parameters. Optimization was run for up to 50,000 iterations, with a mesh and step tolerance of 1 × 10^−6^. To assess uniformity at each iteration, a custom cost function was implemented:

$$\mathrm{CF}=(1-\frac{\text{Mean}(E_{\mathrm{net}})}{\mathrm{Max}(E_{\mathrm{net}})})+\frac{\text{Mean}(E_{\mathrm{net}})}{\text{Mean}(E_{\text{large}})}$$ (5)

The first term represents a ratio between the mean and maximum intensity for the illumination distribution. The second term is a ratio of the mean pixel intensity of the illumination distribution from the desired uFOV divided by the mean pixel intensity of an illumination distribution determined by (3*uFOV*_x_*, 3*uFOV*_y_*). The first term selects for illumination distributions where the average pixel value approaches the maximum value. The ratio of the mean and the maximum pixel value becomes one when all pixels have an intensity equal to the maximum value; this selects for a higher intensity solution and encourages all pixel values to converge on the maximum value. The second term selects for solutions with concentrated illumination distributions. This term penalizes solutions that place illumination outside of the uFOV. Two distinct illumination systems were built based on optimization results. A high-aspect-ratio illumination distribution was optimized by inputting a uFOV of 12 × 4 mm, and a low-aspect-ratio illumination distribution was optimized by inputting a uFOV of 6 × 6 mm.

### Dynamic range studies

The dynamic range of the CMOS camera of the CapCell was assessed using a Stouffer 41-step transparent wedge (optical density, 0.1 to 4) and a white LED lamp through transmission imaging with an integration time of 1/8 s. For each grid step of the transparent wedge, 3 images were averaged to calculate the mean pixel values. Hsp90 fluorescence phantoms were created with tissue-mimicking optical properties and known concentrations of HS-27 (0 to 25 μM based on prior preclinical and clinical studies [25]). Polystyrene beads (Polybead® Microspheres 1.00 μm) were used to mimic tissue optical scattering (μs′  = 20 cm^−1^) of murine mammary tumors [26]. Three images were obtained from each phantom using the high-aspect-ratio platform and average fluorescence intensity was calculated. All data were processed and analyzed in MATLAB. A 95% confidence interval of the linear regression test was used for all tests, with a *P* value less than 0.05 considered statistically significant.

### Cell culture

The murine mammary carcinoma cell line (4T1) was used for the mammary tumor injections in the in vitro organoid culture, in vivo mammary window, and ex vivo biopsies studies. The cell line was obtained from the American Type Culture Collection (ATCC) and was cultured in 90% RPMI-1640 (L-glutamine), 10% FBS, and 1% penicillin–streptomycin under sterile conditions at 37 °C and 5% CO_2_ level. All cells were used within 10 cell passages.

### Animal studies

All animal work was approved by the Duke University Institutional Animal Care and Use Committee (protocol number A038-21-02 and A121-21-06). Animals were housed in an onsite housing facility with ad libitum access to food and water and standard 12-h light/dark cycles.

### Ex vivo biopsy imaging studies

4T1 tumors were grown in the fourth right mammary gland of athymic nude mice (Charles River Laboratories, Raleigh, North Carolina), 6 to 8 weeks old, through an injection of 100 μl (~50,000 cells) of 4T1 cells suspended in serum-free media (RPMI-1640). Once tumors reached a volume of 1 cm^3^, the animal was anesthetized using 2% isoflurane in room air. The skin over the tumor was removed and a 12-gauge biopsy gun was used to acquire 2 biopsies randomly from each tumor. Specimens were stained with either 100 μM HS-27 or HS-217 (*n* = 5 for each group) for 1 min and washed in PBS. All biopsy samples were imaged with the optimized and unoptimized high-aspect-ratio setups at a WD of 25 mm for wide-field and 12.5 mm for high-resolution images. Unoptimized illumination was generated by increasing the distance between the fibers and the sample by 6 mm along the *z* axis. The distance between the sample and the detector was maintained at a constant WD. Both brightfield and fluorescence images were captured with the system. After imaging, biopsy samples were fixed in a 10% formalin solution and prepared for hematoxylin and eosin staining. The hematoxylin and eosin histology slide was scanned on a Zeiss Axio Imager Z2 Upright Microscope. Kolmogorov-Smirnov test was used for the statistical analysis of fluorescence image pixel distributions (survival curves). The Kolmogorov-Smirnov test was performed with the MATLAB Statistics Toolbox, with a *P* value less than 0.05 considered statistically significant.

### Mammary window chamber imaging studies

4T1 mammary tumors were grown in the fourth right mammary gland of athymic nude mice (Charles River, Raleigh, NC), 6 to 8 weeks old and weighing between 20 and 25 g. Once tumors reached approximately 5 × 5 mm, the skin was removed and replaced with a 12-mm-diameter titanium window and a No. 2 glass coverslip. All mice were fasted for at least 2 h before imaging (with water provided) to maintain a normalized metabolic rate for each animal as established previously [9]. Mice were anesthetized through inhalation of 2% v/v isoflurane and maintained at 1% to 1.5% v/v isoflurane for the duration of the imaging procedure. A background fluorescence image was taken using both the low-aspect-ratio and high-aspect-ratio illumination platforms. Animals then received a retro-orbital injection of either 2 mM 2-NBDG, 200 nM Bodipy Fl C16, or 20 mg/kg HS-27. Fluorescence images were acquired at 2-, 10-, 20-, 30-, 40-, and 50-min postinjection using the low-aspect-ratio platform to visualize probe uptake kinetics. At the 60-min time point, a fluorescence image was acquired with both the low- and high-aspect-ratio illumination platforms.

### Imaging of fluorescence beads

Green fluorescence beads (Fluoresbrite® YG Microspheres 45.0 μm) were diluted in a 1:5 ratio with PBS. Twenty microliters of the diluted solution was added to a standard microscope slide. The sample was imaged using the optimized and unoptimized low-aspect-ratio illumination platforms. For this study, nonuniform illumination was created by setting the d*x* and d*y* offsets of the uniform platform to zero. All images were processed with MATLAB 2021a.

### Organoid imaging studies

4T1 murine tumors were excised, rinsed with PBS, and minced. The tissue was then digested to a single-celled suspension in a cocktail medium containing collagenase, DNase, and ROCK (rho kinase) inhibitor on an orbital shaker at 37 °C for 2 h. The digested tissue was strained over a 70-μm filter before centrifugation. If a visible red pellet was seen, erythrocytes were lysed in red blood cell lysis buffer for 5 min and centrifuged again. The pellet was resuspended in ice-cold growth factor reduced Matrigel and plated in 25-μl droplets on cell culture imaging dishes and incubated for 30 min. Organoid medium was then added to the dishes and changed every 2 days. Organoids were passed every 5 to 7 days. Organoids in the 100-μm size range were stained with 200 μM Bodipy FL C16 for 2 h and rinsed with sterile PBS prior to imaging. The specimen was imaged using both optimized and unoptimized low-aspect-ratio illumination platforms, as described for the fluorescent bead study, at an imaging WD of 12.5 mm. All images were processed with MATLAB 2021a.

## Data Availability

All data can be made available upon request to the corresponding authors.

## Supplementary Materials

Supplementary Materials Fig. S1. A custom cost function preferentially selects for uniformity and power density. Both a low-AR and a high-AR illumination profile were optimized using 1 of 3 cost functions: Coefficient of variation, mean–max ratio, or mean–max ratio + efficiency. Each row is normalized to the maximum intensity of the mean–max + efficiency result in the same row. Color bars cover the range of each individual image’s intensity to better visualize uniformity. All optimizations were run for a maximum of 50,000 evaluations with a mesh tolerance of 1 × 10^−6^ and a step tolerance of 1 × 10^−6^. The angular position, θ, was bounded between 30° and 60° and radial distance *R* was bounded between 12.5 and 40 mm. Fig. S2. Repeat optimization produces consistent results. (A) The results of 5 optimization trials to design high-AR uniform illumination. Optimization ran for an average of 93 s and took an average of 32,000 iterations. (B) The results of 5 optimization trials to design low-AR uniform illumination. Optimization ran for an average of 57 s and took an average of 23,000 iterations. All optimizations were run for a maximum of 50,000 evaluations with a mesh tolerance of 1 × 10^−6^ and a step tolerance of 1 × 10^−6^. The angular position, θ, was bounded between 30° and 60° and radial distance *R* was bounded between 12.5 and 40 mm. Fig. S3. The dynamic range of the CapCell encompasses the preclinical and clinical range of Hsp90 imaging. (A) Average pixel intensity was calculated and plotted against known percent light transmission using neutral density filters. All *R*^2^ values are greater than 0.95 with *P* values less than 0.01. (B) Tissue-mimicking HS-27 fluorescence phantoms (0, 2, 5, 10, 15, and 25 μM) were imaged using the CapCell. The relationship between average pixel intensity and phantom concentration is plotted and fit to a linear curve. Representative concentration values of 4T1 murine mammary tumor samples and clinical biopsies acquired from previous clinical studies are included. The resultant *R*^2^ value is 0.98 with a *P* value of less than 0.01. Linear regression was used for statistical analysis. Fig. S4. Flat field correction applied to the representative optimized biopsy and unoptimized biopsy images. A flat field correction is applied to the biopsy images by dividing the fluorescent standard images from the fluorescent biopsy images pixel-by-pixel. The top row shows the biopsy image acquired from the optimized setup (left) and unoptimized setup (right) without flat field correction. The bottom row shows the biopsies imaged with the optimized setup (left) and unoptimized setup (right) with flat field correction. The flat field correction gives little change in the overall fluorescence features of the biopsy samples.
