# Supplementary material for: Adaptive Design of Fluorescence Imaging Systems for Custom Resolution, Fields of View, and Geometries
Source: BME Front. 2023 Jan 13;4:0005. doi: 10.34133/bmef.0005 (PMC10521686; doi:10.34133/bmef.0005)
Supplement: Supplementary Materials — Fig. S1. A custom cost function preferentially selects for uniformity and power density. Both a low-AR and a high-AR illumination profile were optimized using 1 of 3 cost functions: Coefficient of variation, mean–max ratio, or mean–max ratio + efficiency. Each row is normalized to the maximum intensity of the mean–max + efficiency result in the same row. Color bars cover the range of each individual image’s intensity to better visualize uniformity. All optimizations were run for a maximum of 50,000 evaluations with a mesh tolerance of 1 × 10^−6^ and a step tolerance of 1 × 10^−6^. The angular position, θ, was bounded between 30° and 60° and radial distance R was bounded between 12.5 and 40 mm. Fig. S2. Repeat optimization produces consistent results. (A) The results of 5 optimization trials to design high-AR uniform illumination. Optimization ran for an average of 93 s and took an average of 32,000 iterations. (B) The results of 5 optimization trials to design low-AR uniform illumination. Optimization ran for an average of 57 s and took an average of 23,000 iterations. All optimizations were run for a maximum of 50,000 evaluations with a mesh tolerance of 1 × 10^−6^ and a step tolerance of 1 × 10^−6^. The angular position, θ, was bounded between 30° and 60° and radial distance R was bounded between 12.5 and 40 mm. Fig. S3. The dynamic range of the CapCell encompasses the preclinical and clinical range of Hsp90 imaging. (A) Average pixel intensity was calculated and plotted against known percent light transmission using neutral density filters. All R2 values are greater than 0.95 with P values less than 0.01. (B) Tissue-mimicking HS-27 fluorescence phantoms (0, 2, 5, 10, 15, and 25 μM) were imaged using the CapCell. The relationship between average pixel intensity and phantom concentration is plotted and fit to a linear curve. Representative concentration values of 4T1 murine mammary tumor samples and clinical biopsies acquired from previous clinical studies are included. Th [file bmef.0005.f1.docx]

SUPPLEMENTARY MATERIALS


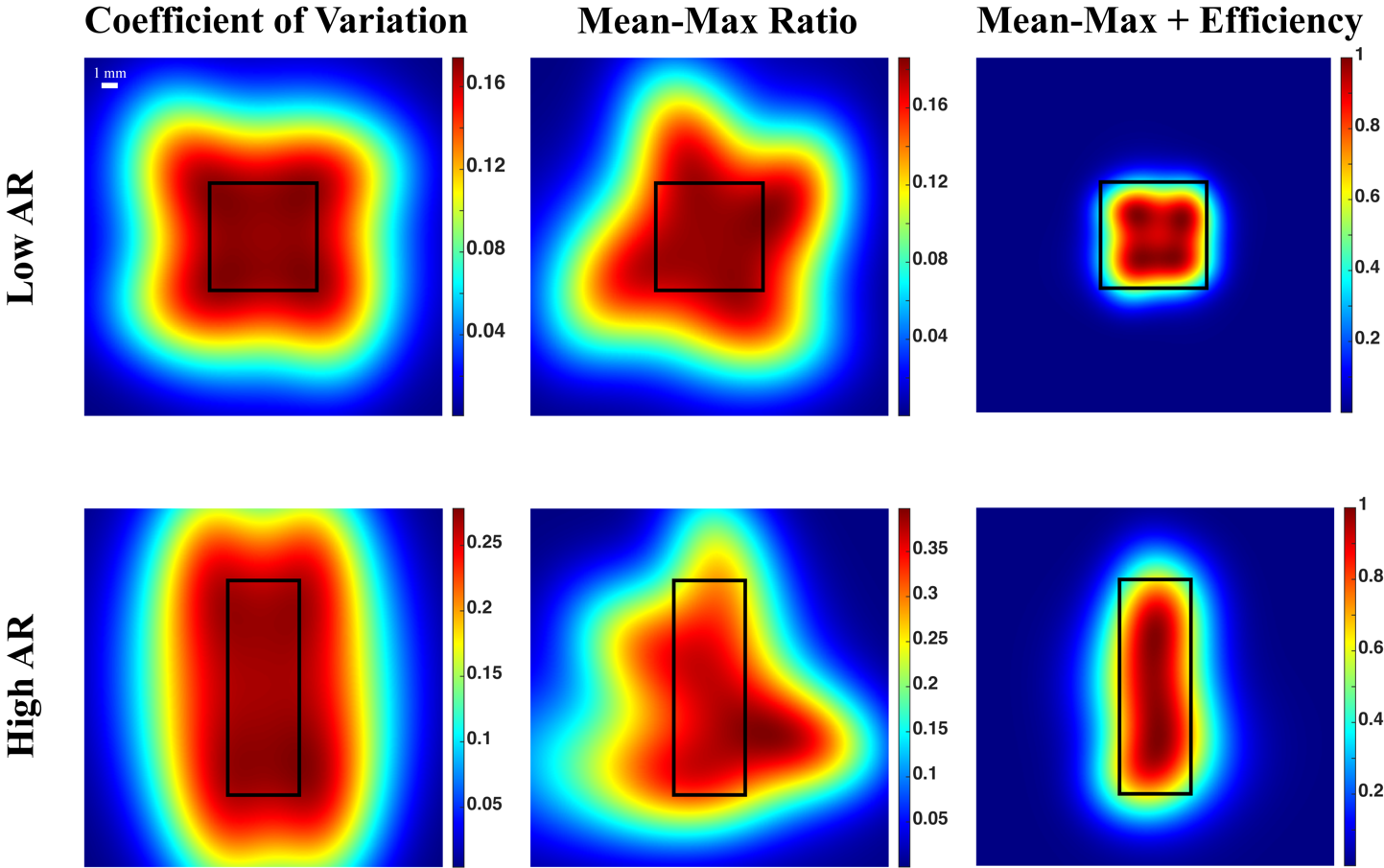


**Supplemental Fig. 1:** A custom cost function preferentially selects for uniformity and power density. Both a low AR and high AR illumination profile were optimized using one of three cost functions: Coefficient of variation, mean-max ratio, or mean-max ratio + efficiency. Each row is normalized to the maximum intensity of the mean-max+efficiency result in the same row. Color bars cover the range of each individual image’s intensity to better visualize uniformity. All optimizations were run for a maximum of 50,000 evaluations with a mesh tolerance of 1x10^-6 and a step tolerance of 1x10^-6. The angular position, theta, was bounded between 30 and 60 degrees and radial distance R was bounded between 12.5- and 40-mm.


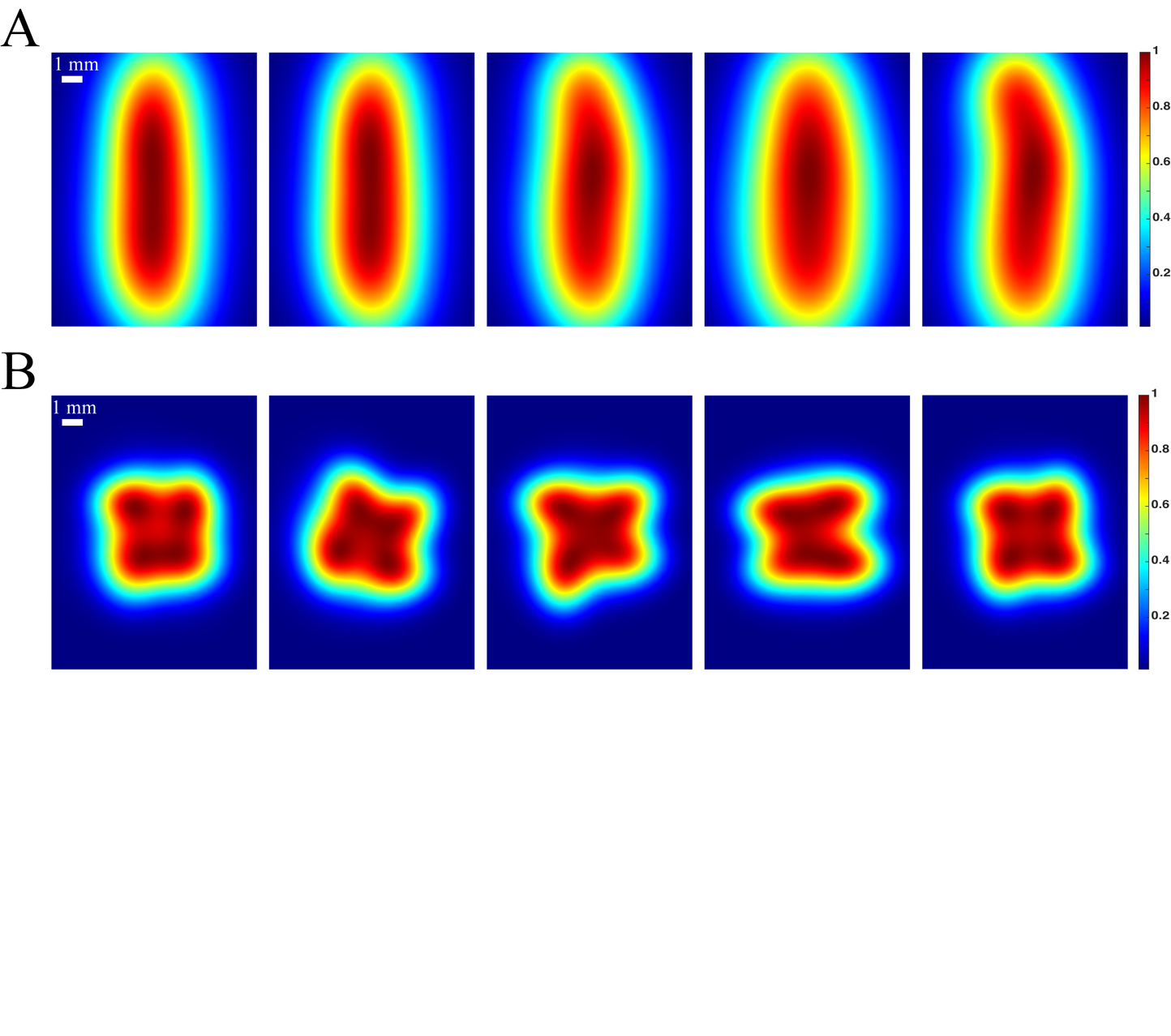


**Supplemental Fig. 2:** Repeat optimization produces consistent results. **A)** The results of five optimization trials to design high AR uniform illumination. Optimization ran for an average of 93 s and took an average of 32,000 iterations. **B)** The results of five optimization trials to design low AR uniform illumination. Optimization ran for an average of 57 s and took an average of 23,000 iterations. All optimizations were run for a maximum of 50,000 evaluations with a mesh tolerance of 1x10^-6 and a step tolerance of 1x10^-6. The angular position, theta, was bounded between 30 and 60 degrees and radial distance R was bounded between 12.5- and 40-mm.


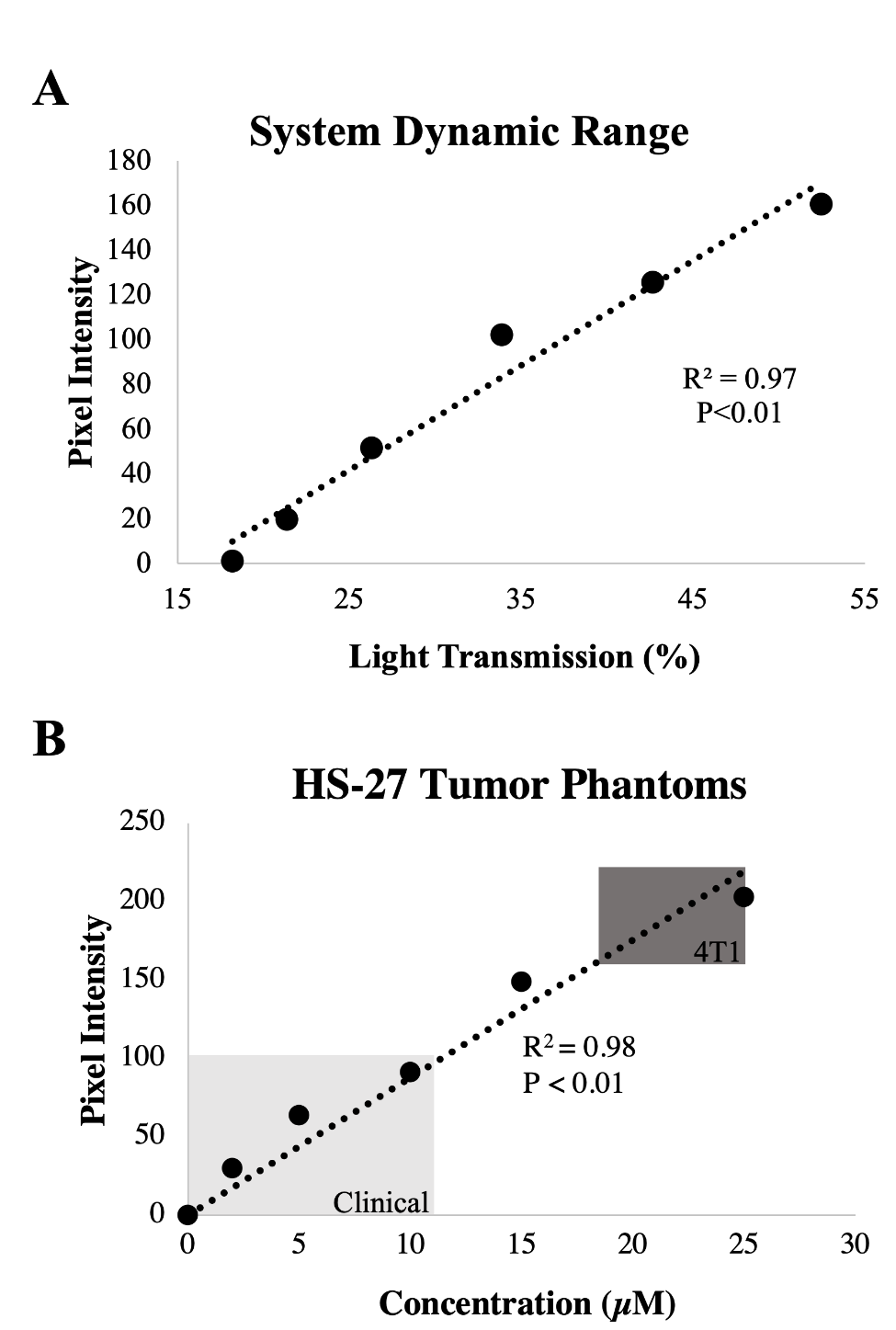


**Supplemental Fig. 3:** The dynamic range of the CapCell encompasses the preclinical and clinical range of Hsp90 imaging. **A)** Average pixel intensity was calculated and plotted against known percent light transmission using neutral density filters. All R^2^ values are greater than 0.95 with p-values less than 0.01. **B)** 0, 2, 5, 10, 15, and 25 μM tissue-mimicking HS-27 fluorescence phantoms were imaged using the CapCell. The relationship between average pixel intensity and phantom concentration is plotted and fit to a linear curve. Representative concentration values of 4T1 murine mammary tumor samples and clinical biopsies acquired from previous clinical studies are included. The resultant R^2^ value is 0.98 with a p-value of less than 0.01. Linear regression was used for statistical analysis.


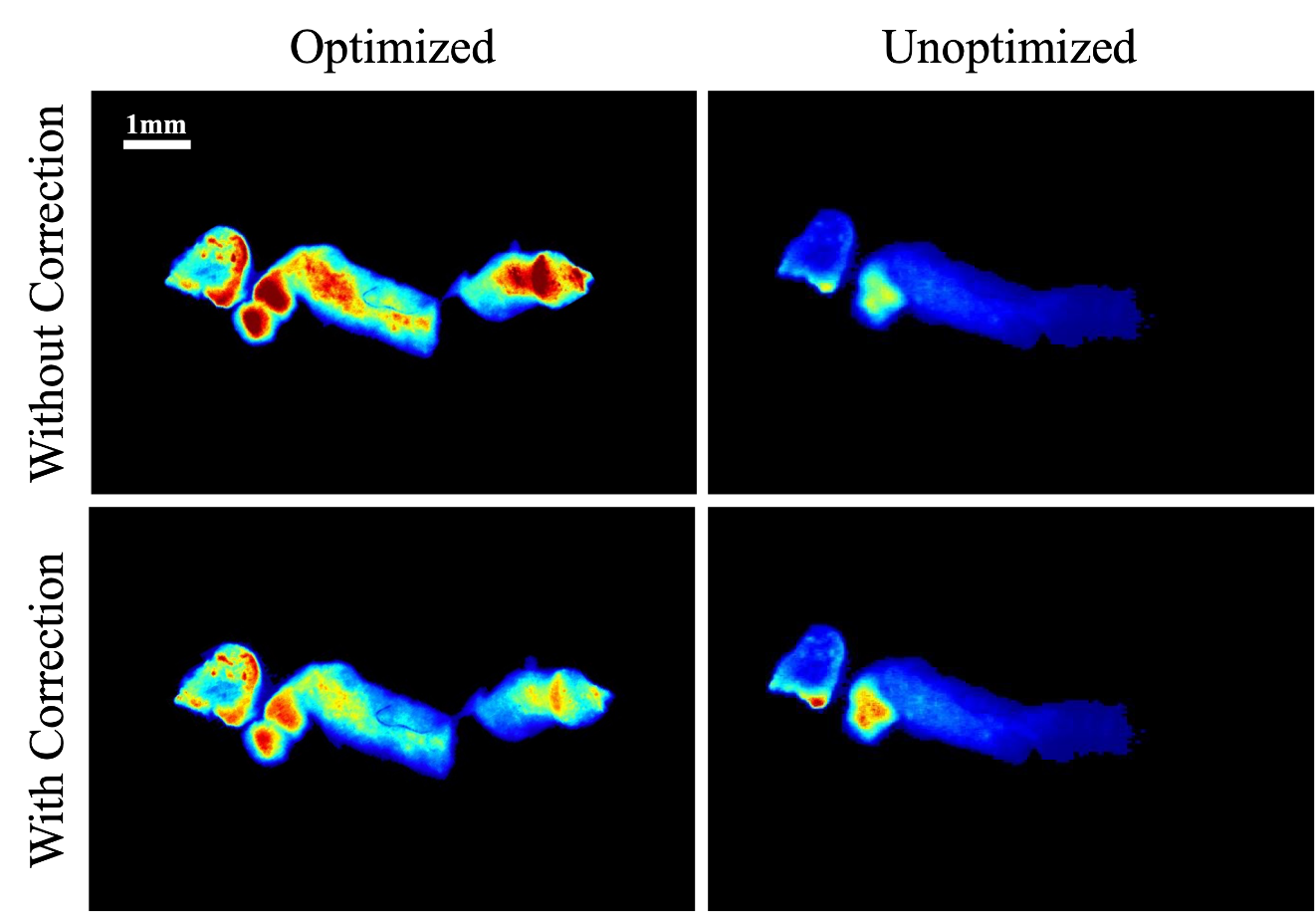


**Supplemental Fig. 4:** Flat field correction applied to the representative optimized biopsy and unoptimized biopsy images. A flat field correction is applied to the biopsy images by dividing the fluorescent standard images from the fluorescent biopsy images pixel-by-pixel. The top row shows the biopsies image acquired from the optimized setup (left) and unoptimized setup (right) without flat field correction. The bottom row shows the biopsies imaged with the optimized setup (left) and unoptimized setup (right) with flat field correction. The flat field correction gives little change in the overall fluorescence features of the biopsy samples.
